# A 23 bp *cyp51A* Promoter Deletion Associated With Voriconazole Resistance in Clinical and Environmental Isolates of *Neocosmospora keratoplastica*

**DOI:** 10.3389/fmicb.2020.00272

**Published:** 2020-03-31

**Authors:** Jasper Elvin James, Erwin Lamping, Jacinta Santhanam, Trudy Jane Milne, Mohd Fuat Abd Razak, Latiffah Zakaria, Richard David Cannon

**Affiliations:** ^1^Biomedical Science Programme, Faculty of Health Sciences, Universiti Kebangsaan Malaysia, Kuala Lumpur, Malaysia; ^2^Faculty of Dentistry, Sir John Walsh Research Institute, University of Otago, Dunedin, New Zealand; ^3^Bacteriology Unit, Institute for Medical Research, National Institute of Health, Setia Alam, Malaysia; ^4^School of Biological Sciences, Universiti Sains Malaysia, Penang, Malaysia

**Keywords:** azole, Cyp51A, *Neocosmospora*, *Fusarium*, FSSC, sterol regulatory element

## Abstract

In the fungal pathogen *Aspergillus fumigatus*, resistance to azole antifungals is often linked to mutations in *CYP51A*, a gene that encodes the azole antifungal drug target lanosterol 14α-demethylase. The aim of this study was to investigate whether similar changes could be associated with azole resistance in a Malaysian *Fusarium solani* species complex (FSSC) isolate collection. Most (11 of 15) clinical FSSC isolates were *Neocosmospora keratoplastica* and the majority (6 of 10) of environmental isolates were *Neocosmospora suttoniana* strains. All 25 FSSC isolates had high minimum inhibitory concentrations (MICs) for itraconazole and posaconazole, low MICs for amphotericin B, and various (1 to >32 mg/l) voriconazole susceptibilities. There was a tight association between a 23 bp *CYP51A* promoter deletion and high (>32 mg/l) voriconazole MICs; of 19 FSSC strains sequenced, nine isolates had voriconazole MICs > 32 mg/l, and they all contained the 23 bp *CYP51A* promoter deletion, although it was absent in the ten remaining isolates with low (≤12 mg/l) voriconazole MICs. Surprisingly, this association between voriconazole resistance and the 23 bp *CYP51A* promoter deletion held true across species boundaries. It was randomly distributed within and across species boundaries and both types of FSSC isolates were found among environmental and clinical isolates. Three randomly selected *N. keratoplastica* isolates with low (≤8 mg/l) voriconazole MICs had significantly lower (1.3–7.5 times) *CYP51A* mRNA expression levels than three randomly selected *N. keratoplastica* isolates with high (>32 mg/l) voriconazole MICs. *CYP51A* expression levels, however, were equally strongly induced (~6,500-fold) by voriconazole in two representative strains reaching levels, after 80 min of induction, that were comparable to those of *CYP51B*. Our results suggest that FSSC isolates with high voriconazole MICs have a 23 bp *CYP51A* promoter deletion that provides a potentially useful marker for voriconazole resistance in FSSC isolates. Early detection of possible voriconazole resistance is critical for choosing the correct treatment option for patients with invasive fusariosis.

## Introduction

There is heightened concern about invasive mould infections (IMIs) as a consequence of the increasing number of immunocompromised patients (Lass-Flörl and Cuenca-Estrella, 2017; Rotjanapan et al., 2018). Fusaria are among the most frequent cause of IMIs after aspergilli (Nucci and Anaissie, 2007). They are also significant plant pathogens, causing severe vascular wilt and root rot disease in agriculturally important crops (Coleman, 2016; Kazan and Gardiner, 2018; Urbaniak et al., 2018). The *Fusarium solani* species complex (FSSC) comprises more than 60 species and accounts for ~60% of fusariosis cases worldwide (O'Donnell et al., 2010; Schroers et al., 2016). Fusariosis ranges from localised skin, nail, and eye lesions to disseminated infections (Al-Hatmi et al., 2018). In Singapore, an outbreak of contact lens associated keratitis caused by FSSC species occurred in 2005 involving 66 patients (Jureen et al., 2008). An epidemiological study in Japan, from 1998 to 2015, found that FSSC species accounted for 72.6% of all fusariosis cases, of which 77.8% were invasive fusariosis (IF) (Muraosa et al., 2017). An analysis of a collection of environmental *Fusarium* isolates from the Malaysian highlands found that 66.1% of 1,449 isolates belonged to the FSSC (Manshor et al., 2012), highlighting their predominance in Malaysia. Reports of clinical FSSC isolates in Malaysia are, however, rare. The first confirmed report was of a patient diagnosed with *Fusarium* keratitis in 1981 (Singh et al., 1981). Most other studies since then were reports of antifungal drug susceptibility testing of a few *Fusarium* isolates (Santhanam et al., 2008; Tzar et al., 2013, 2014, 2016). Historically, species of the FSSC were simply referred to as *Fusarium solani*. However, a *Fusarium* keratitis outbreak in the United States (Chang et al., 2006) changed that view through the application of multilocus sequence typing (MLST). “*F. solani*” species were divided into different “clades” based on translation elongation factor 1-alpha (*TEF1-*α) and RNA polymerase II (*RPB2*) sequences. The internal transcribed spacer (ITS) regions alone were insufficient to differentiate between species of the FSSC (O'Donnell and Cigelnik, 1997). More recently, Sandoval-Denis and Crous proposed renaming species of the FSSC because they actually belong to the related *Neocosmospora* genus (Sandoval-Denis and Crous, 2018); *Fusarium petroliphilium* (FSSC 1 clade) was renamed *N. petroliphila, F. keratoplasticum* (FSSC 2 clade) was renamed *N. keratoplastica, F. falciforme* (FSSC 3 clade) was renamed *N. falciformis*, and strains of the FSSC 7, 20 and 43 clades were named *N. gamsii, N. suttoniana*, and *N. catenate*, respectively (Sandoval-Denis and Crous, 2018).

Most *Fusarium* species have low susceptibilities to the majority of azole antifungals (Tupaki-Sreepurna et al., 2017; Rotjanapan et al., 2018; Herkert et al., 2019) and patients with IFs have high mortality rates (Esnakula et al., 2013; Silva et al., 2013; Okada et al., 2018). Voriconazole (VRC) and amphotericin B (AMB) are the recommended treatment options for localised infections and IFs (Efe İris et al., 2016; Okada et al., 2018). This is despite the fact that most FSSC isolates show relatively low VRC susceptibilities with the majority (369 of 555; 66%) exhibiting a minimum growth inhibitory concentration for VRC (MIC_VRC_) of ≥8 mg/l (Espinel-Ingroff et al., 2016). Unlike most other Ascomycetes that have only one *CYP51* gene, moulds of the Pezizomycotina clade have two *CYP51* paralogues (*CYP51A* and *CYP51B*) (Brillowska-Dabrowska et al., 2015; Ruan et al., 2017; Abastabar et al., 2019), and *Fusarium* and *Neocosmospora* species also have a third *CYP51* paralogue, *CYP51C* (Liu et al., 2011; Fan et al., 2013). In *A. fumigatus*, modifications of *CYP51A* are major contributors to azole resistance (Chowdhary et al., 2017). However, azole resistance mechanisms of species of the FSSC remain largely unknown.

The roles of the three *CYP51* paralogues in growth, ascospore formation, azole resistance, and pathogenicity have been explored to some extent in the related plant fungal pathogen *Fusarium graminearum* (Becher et al., 2010; Liu et al., 2011; Fan et al., 2013). Although *CYP51B* accounts for most of the lanosterol 14α-demethylase activity under normal growth conditions, deletion of *CYP51B* had no effect on growth or viability because upregulation of *CYP51A* compensated for the loss of its activity (Fan et al., 2013). However, *CYP51B* deletion reduced ascospore formation and caused an increase in eburicol and 14-methylated sterol content in membranes (Fan et al., 2013). *CYP51A* was also upregulated in response to azole inhibition, and its deletion caused a 30-fold increase in azole sensitivities (Fan et al., 2013). It appears that, as in *A. fumigatus, CYP51A* is mainly responsible for the observed azole susceptibilities in *F. graminearum* (Liu et al., 2011; Fan et al., 2013). Cyp51C had no lanosterol 14α-demethylase activity but it was required for invasion of plant tissue (Fan et al., 2013).

The increasing incidence of IFs (Lortholary et al., 2010; Nucci et al., 2013; Tortorano et al., 2014) and reports of azole resistance caused by the widespread use of azole fungicides in agriculture (Chowdhary et al., 2013; Faria-Ramos et al., 2014; Vaezi et al., 2018) prompted us to study possible azole resistance mechanisms in *Neocosmospora* species of the FSSC.

## Materials and Methods

### Fungal Isolates

Fifteen *Neocosmospora* clinical FSSC isolates were obtained from the Hospital Canselor Tuanku Muhriz UKM & Institute for Medical Research, Malaysia. They had originally been collected from nail, skin, corneal scraping, and blood as part of routine diagnostic procedures. No identifying data from any humans were obtained or utilised in this study. A further 10 were environmental isolates from soil and plant debris (Table 1). All isolates were presumptively identified as *Neocosmospora* species based on conidia morphology (Leslie and Summerell, 2008). The isolates were grown on potato dextrose agar, PDA (Merck & Co., Kenilworth, USA) with incubation at 28°C for 4 to 7 days.

**Table 1:** List of 15 clinical and 10 environmental FSSC isolates from Malaysia, their MLST clade and GenBank accession numbers for *TEF1-*α and *RPB2*.

**Isolate**	**Source^**a**^**	**Species**	**MLST clade**	**GenBank accession number**	
				***TEF1-α***	***RPB2***
**CLINICAL ISOLATES**					
Np667	Eye	*N. petroliphila*	1-b	MN178239	MN263125
Nk620	Skin	*N. keratoplastica*	2-a	MN178238	MN263124
Nk2781	Nail	*N. keratoplastica*	2-a	MN178234	MN263120
Nk2309	Nail	*N. keratoplastica*	2-f	MN178231	MN263117
Nk553	Skin	*N. keratoplastica*	2-h	MN178237	MN263123
Nk2353	Nail	*N. keratoplastica*	2-h	MN178232	MN263118
Nk994	Nail	*N. keratoplastica*	2-h	MN178240	MN263126
Nk0168	Blood	*N. keratoplastica*	2-h	MN178228	MN263114
Nk2622	Nail	*N. keratoplastica*	2-h	MN178233	MN263119
Nk1049	Nail	*N. keratoplastica*	2-h	MN178241	MN263127
Nk1931	Nail	*N. keratoplastica*	2-k	MN178230	MN263116
Nk1930	Nail	*N. keratoplastica*	2-k	MN178229	MN263115
Nf0020	Eye	*N. falciformis*	3+4-k	MN178227	MN263113
Nf541	Blood	*N. falciformis*	3+4-oo	MN178236	MN263122
Ns263	Eye	*N. suttoniana*	20-c	MN178235	MN263121
**ENVIRONMENTAL ISOLATES**					
NkDI17	Grass	*N. keratoplastica*	2-a	MN178221	MN263107
NkDir61	Grass	*N. keratoplastica*	2-a	MN178225	MN263111
Nf4225	Tobacco	*N. falciformis*	3+4+k	MN178212	MN263098
Nf4290	Straw compost	*N. falciformis*	3+4+k	MN178215	MN263101
Nf4325	Honeydew	*N. falciformis*	3+4+k	MN178217	MN263103
Ns3769	Coconut tree	*N. suttoniana*	20-c	MN178207	MN263093
Ns3784	Mangrove	*N. suttoniana*	20-c	MN178209	MN263094
Ns3873	Grass	*N. suttoniana*	20-c	MN178208	MN263095
Ns3924	Sugarcane	*N. suttoniana*	20-c	MN178210	MN263096
Ns4279	Dragon fruit	*N. suttoniana*	20-c	MN178214	MN263100

a *The source for environmental isolates describes the type of plant environment from which the soil samples were collected*.

### Molecular Identification and Antifungal Susceptibility Testing

*Neocosmospora* isolates were identified from their *TEF1-*α and *RPB2* gene sequences (GenBank accession numbers are listed in Table 1) using the *Fusarium* MLST database (O'Donnell et al., 2008, 2012). Etest susceptibility testing was performed on 1.5% (w/v) agar (Merck & Co., Kenilworth, USA) plates containing 10.4 g/l RPMI 1640 medium R6504 (Sigma-Aldrich, St. Louis, USA), 2% (w/v) glucose (Merck & Co., Kenilworth, USA), and 165 mM MOPS (pH 7.0) (Sigma-Aldrich, St. Louis, USA). Etest strips for itraconazole (ITC), posaconazole (POS), VRC, and AMB were purchased from Biomerieux, Marcy I′Etoile (France). Conidial suspensions (~1–5 × 10^6^ cfu/ml) were prepared according to the CLSI M38-A2 protocol (CLSI, 2008) with a slight modification. The mycelia of a 2-week old culture grown on PDA at 28°C was flooded with ~2 ml sterile 0.85% NaCl and scraped off using a pipette tip followed by filtration through a sterile double layer of gauze to remove large hyphae fragments. The densities of conidial cell suspensions were adjusted to an optical density (OD) of 0.16 measured at 530 nm. Susceptibilities were measured after inoculation of RPMI agar plates with a sterile cotton swab soaked in the conidial suspension, placing Etest strips on top of the cell layer, and incubating the plates at 35°C for 46–50 h. *A. fumigatus* ATCC 204305 and *Candida parapsilosis* ATCC 22019 were used as susceptible controls. The MICs were determined according to the Etest reading guide. Because clinical antifungal breakpoints have not yet been established for FSSC species, categorising individual isolates as susceptible, susceptible-dose-dependent, or resistant was not possible. Instead, we used the epidemiological cut-off values (ECVs) determined for FSSC species (Espinel-Ingroff et al., 2016) as a guide to interpret antifungal drug susceptibilities.

### Genomic DNA Extraction

Cells of *Neocosmospora* isolates grown for a week on PDA plates at 28°C were collected in 1.5 ml 0.85% NaCl solution and homogenised in a 2 ml microcentrifuge tube with a mini pestle BioMasher-II (OPTIMA Inc., Itabashi-ku, Japan). The homogenised cell suspension was harvested by centrifugation at 15,339 g for 5 min, and genomic DNA (gDNA) was extracted from the cell pellet with a DNeasy Plant Extraction Kit (QIAGEN Inc., Valencia, USA) according to the manufacturer's instructions.

### Amplification of *CYP51* Genes

DNA oligomer primers used in this study are listed in Supplementary Table S1. Primers were initially designed using the only *Neocosmospora* genome sequence available at the time, the teleomorph *Nectria haematococca* mpVI 77-13-4 (Coleman et al., 2009). *N. haematococca CYP51A, CYP51B*, and *CYP51C* were identified with BLAST searches using the Cyp51A, Cyp51B, and Cyp51C (GenBank accession nos. XP_011321548, XP_011316750, and XP_011325340, respectively) (Cuomo et al., 2007) sequences of *F. graminearum* PH-1 as queries. *CYP51A, CYP51B*, and *CYP51C*, including parts of their 5′-upstream and 3′-downstream ORF sequences and parts of the housekeeping genes β-actin (*ACT1*) and GAPDH (*GPD1*), were amplified by PCR using gDNA extracts of *N. keratoplastica* Nk553, Nk2309, and Nk2781 as DNA templates and sequencing the DNA fragments. PCR amplifications were performed with HotStarTaq Master Mix (QIAGEN Inc., Valencia, USA) using 35 cycles of denaturation at 94°C for 30 s, annealing at 55°C for 30 s and extension at 72°C for 1 min on a T100™ thermal cycler (Bio-Rad, Hercules, USA). DNA sequences were submitted to GenBank and accession numbers for *CYP51A, CYP51B, CYP51C, ACT1*, and *GPD1* are listed in Supplementary Table S2. *N. keratoplastica CYP51A, CYP51B, CYP51C, ACT1*, and *GPD1* sequences were used to design species specific primers for qPCR amplification (Supplementary Table S1).

### Phylogenetic Tree of *N. keratoplastica CYP51* Orthologs

Introns of the *CYP51* paralogues of *N. keratoplastica* Nk2781 were verified by PCR amplification and DNA sequencing of the cDNA. Protein alignments of related sequences were created with CLUSTALW (Thompson et al., 1994) and manually corrected if necessary. Phylogenetic inferences using two independent algorithms, Maximum Parsimony (MP) and Maximum Likelihood (ML), with 1,000 bootstrap (BS) replicates were performed using the Molecular Evolutionary Genetics Analysis (MEGA) software version 10.0.5 (Kumar et al., 2018). GenBank accession numbers for the Cyp51/Erg11 sequences that were used for the phylogenetic analysis are listed in Supplementary Table S3.

### Total RNA Extraction

Conidial suspensions (10 μl of ~1 ×10^8^ cfu/ml) of three *N. keratoplastica* isolates with MIC_VRC_s > 32 mg/l (Nk2309, Nk2781, NkDI17) and three with MIC_VRC_s ≤ 8 mg/l (NkDir61, Nk553, Nk994) were used to inoculate 50 ml potato dextrose broth (PDB) (Merck & Co., Kenilworth, USA) and incubated at 30°C for 21 h with shaking at 200 rpm. Logarithmic cells were harvested by filtration through a glass fibre round filter No. 6 (Schleicher & Schuell BioScience GmbH, Dassel, Germany) using a vacuum manifold and washed once with ~5 ml ice-cold distilled water. The “cell cake” was scraped off the filter with a scalpel, quickly transferred into a 1.5 ml microcentrifuge tube, snap frozen in liquid nitrogen, and stored at −80°C. Total RNA was extracted from frozen cell pellets (~100 mg) with a hot-phenol extraction protocol. In short, frozen cell pellets were dropped into a 15 ml Corex tube containing a mixture of 1 ml acid phenol (saturated with SAB buffer; 50 mM sodium acetate, 10 mM EDTA, pH 5.0), 2 ml SAB buffer, 100 μl 10% SDS, and ~1 g 0.5 mm acid-washed zirconia beads (BioSpec Products, Bartlesville, USA), at 65°C. The cells were broken and RNA released into the water phase by five cycles of 30 s thorough vortexing with 1.5 min incubation at 65°C in between each cycle. Liquid phases were separated by centrifugation at 10,000 g for 10 min and traces of phenol in the ~2 ml upper phase removed by chloroform extraction. Total RNA was ethanol precipitated, harvested by centrifugation at 30,000 g for 30 min, air dried for 10 min, and resuspended in 200 μl RNAse-free water. Traces of gDNA were removed by DNase treatment of samples with a PureLink DNase kit (Invitrogen Inc., Carlsbad, USA). Spectrophotometrically determined RNA concentrations and RNA integrity of total RNA extracts were confirmed by RNA gel electrophoresis (Supplementary Figure S1).

### qPCR Quantification of mRNA Expression Levels

First strand cDNA was synthesised from 1 μg total RNA using the SuperScript IV VILO Master Mix (Invitrogen Inc., Carlsbad, USA) following the manufacturer's instructions. Quantification of *CYP51A, CYP51B*, and *CYP51C* cDNA by real-time PCR using Fast SYBR Green Master Mix (Applied Biosystems Inc., Foster City, USA) was performed with the QuantStudio 6 Flex Real-Time PCR System (Applied Biosystems Inc., Foster City, USA). The qPCR assay contained 1X Fast SYBR Green Master Mix, 400 nM each of forward and reverse primer, and 5 ng of cDNA template. Thermal cycling steps included a 95°C denaturation step for 20 s followed by 40 cycles of denaturation at 95°C for 3 s and DNA synthesis at 60°C for 30 s. Four 10-fold serial dilutions (5–0.005 ng) of first strand cDNA templates were used to determine the linear amplification range and the amplification efficiencies for each qPCR primer pair. A H_2_O negative control without cDNA template and a -RT control were included. An average quantification cycle (Cq) value for each sample was calculated from two technical replicates. mRNA transcript levels (2^−Δ*Cq*^) were normalised to the referenced housekeeping genes *ACT1* and *GPD1*. The fold-change of *CYP51A* mRNA levels relative to those in the strain with the lowest MIC_VRC_ (1 mg/l), Nk994, was calculated using the ΔΔCq method (2^−ΔΔ*Cq*^) (Livak and Schmittgen, 2001). Fold-change values between zero and one were expressed as fold-regulation [−1/(2^−ΔΔ*Cq*^)].

### *CYP51A, CYP51B*, and *CYP51C* mRNA Expression Levels in Cells Grown in the Presence of VRC for 4 h or Exposed to Various Stress Conditions for 20 min

Total RNA was extracted from 50 ml logarithmic phase cell cultures of Nk2781 or Nk994 (i.e., cells were grown in PDB for 21 h at 30°C, as described above) that had been exposed for 0, 20, 40, 80, or 240 min to 16 mg/l VRC, or for 20 min to various stress conditions: 2 mM H_2_O_2_, 500 mM NaCl, pH 7.0 (165 mM MOPS), 37°C. A no-stress control was included by adding an equivalent volume of sterile H_2_O to the cells.

## Results

### Antifungal Susceptibilities of 15 Clinical and 10 Environmental *Neocosmospora* Isolates

Thirteen of the 25 FSSC isolates were classified as *N. keratoplastica* (FSSC 2), five as *N. falciformis* (FSSC 3+4), six as *N. suttoniana* (FSSC 20), and one as *N. petroliphila* (FSSC 1) (Table 1). As expected (Espinel-Ingroff et al., 2016; Herkert et al., 2019), all 25 FSSC isolates had very high (>32 mg/l) MICs for the long-tailed azoles, ITC, and POS. Initially, the susceptibility of 15 clinical FSSC isolates to ITC was measured and all isolates showed very high MIC_ITC_s (>32 mg/l), and so we predicted the same or higher MIC_ITC_s in FSSC environmental isolates. Therefore, we measured the susceptibility of 10 environmental isolates to another long-tailed azole, POS, instead. All 25 FSSC isolates showed moderately low MIC_AMB_s (≤3 mg/l) (Tables 2, 3, Supplementary Figure S2). However, the *in vitro* VRC susceptibilities divided into two groups: 14 group I isolates with MIC_VRC_s ≤ 12 mg/l and 11 group II isolates that did not respond to VRC at all (MICs > 32 mg/l; Table 2). All clinical (1) and environmental (5) *N. suttoniana* (FSSC MLST clade 20-c) isolates had high MIC_VRC_s (>32 mg/l). The five *N. falciformis* isolates divided into two groups with the three environmental isolates belonging to group I (MICs of 3 or 4 mg/l) and the two clinical isolates to group II (MICs > 32 mg/l), respectively. The majority (10 of 13; 77%) of *N. keratoplastica* isolates and the only *N. petroliphila* clinical isolate were group I isolates with MIC_VRC_s ranging from 1 to 12 mg/l. However, two clinical *N. keratoplastica* isolates (Nk2781 and Nk2309) and one environmental isolate (NkDI17) were group II isolates with high MIC_VRC_s > 32 mg/l (Tables 2, 3).

**Table 2:** Etest antifungal susceptibilities (MICs) of 15 clinical and 10 environmental FSSC isolates.

**Isolate code**	**Species name**	**MIC (mg/l)**^**a, b**^			**VRC susceptibility group^c^**
		**ITC**	**VRC**	**AMB**	
**CLINICAL ISOLATES**					
Np667	*N. petroliphila*	>32	2	0.006	
Nk994	*N. keratoplastica*	>32	1	0.75	
Nk553	*N. keratoplastica*	>32	1.5	1	
Nk620	*N. keratoplastica*	>32	3	0.38	
Nk1931	*N. keratoplastica*	>32	3	0.5	Group I
Nk1049	*N. keratoplastica*	>32	3	2	
Nk1930	*N. keratoplastica*	>32	4	1	
Nk2353	*N. keratoplastica*	>32	4	0.5	
Nk0168	*N. keratoplastica*	>32	6	2	
Nk2622	*N. keratoplastica*	>32	12	0.75	
Nk2309	*N. keratoplastica*	>32	>32	1	
Nk2781	*N. keratoplastica*	>32	>32	3	
Nf0020	*N. falciformis*	>32	>32	0.006	Group II
Nf541	*N. falciformis*	>32	>32	0.023	
Ns263	*N. suttoniana*	>32	>32	0.19	
		**POS**	**VRC**	**AMB**	
**ENVIRONMENTAL ISOLATES**					
Nf4225	*N. falciformis*	>32	3	0.75	Group I
Nf4290	*N. falciformis*	>32	3	0.75	
Nf4325	*N. falciformis*	>32	4	0.75	
NkDir61	*N. keratoplastica*	>32	8	0.75	
NkDI17	*N. keratoplastica*	>32	>32	1	Group II
Ns3769	*N. suttoniana*	>32	>32	0.75	
Ns3784	*N. suttoniana*	>32	>32	0.5	
Ns3873	*N. suttoniana*	>32	>32	3	
Ns3924	*N. suttoniana*	>32	>32	3	
Ns4279	*N. suttoniana*	>32	>32	2	

a *ECVs for ITC, POS, and VRC are 32 mg/l and for AMB 8 mg/l (Espinel-Ingroff et al., 2016)*.

b *AMB and VRC MICs are values for 100% growth inhibition and the listed ITC and POS MICs are the values for 80% growth inhibition*.

c *Group I: VRC MICs ≤ 12 mg/l; Group II: VRC MICs > 32 mg/l*.

**Table 3:** Antifungal susceptibilities (MICs) of 24 FSSC isolates.

**Strain^a^**	**Antifungal**	**MIC (mg/l)**^**b, c, d**^			**Frequency (%) of MICs > 32 mg/l**
		**Range**	**GM**	**MIC_50_^e^**	
*N. keratoplastica*	ITC/POS	>32	>32	ND	100
(*n* = 13)	VRC	1–>32	5.97	6	23
	AMB	0.38–3	0.95	1	–
*N. suttoniana*	ITC/POS	>32	>32	ND	100
(*n* = 6)	VRC	>32	>32	ND	100
	AMB	0.19–3	1.04	0.75	–
*N. falciformis*	ITC/POS	>32	>32	ND	100
(*n* = 5)	VRC	3–>32	8.19	4	40
	AMB	0.006–0.75	0.09	0.75	–

a *N. petroliphila is excluded because there was only one isolate collected*.

b *ECVs for ITC, POS, and VRC are 32 mg/l and for AMB 8 mg/l (Espinel-Ingroff et al., 2016)*.

c *AMB and VRC MICs are values for 100% growth inhibition, and the listed ITC and POS MICs are the values for 80% growth inhibition*.

d *GM: geometric mean*.

e *ND: could not be determined because of the lack of any growth inhibition within the test range*.

### *N. keratoplastica CYP51A, CYP51B*, and *CYP51C*

**Figure 1:**
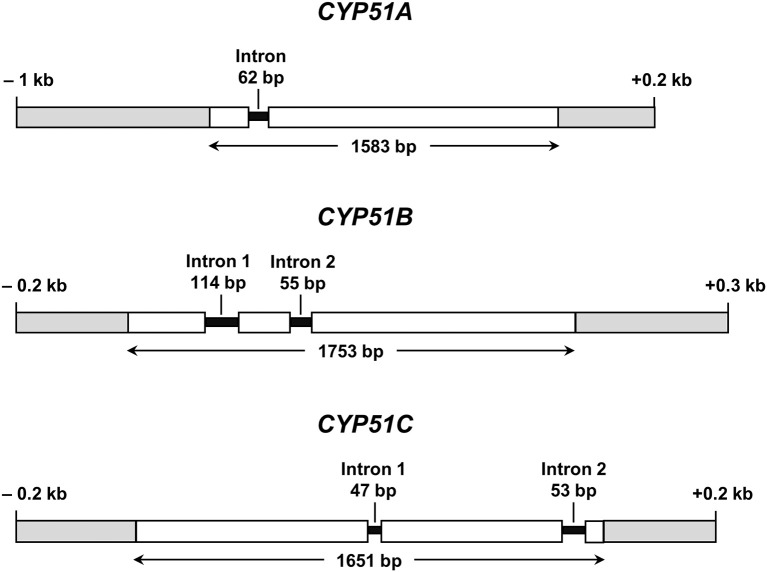
Graphical representations of *N. keratoplastica CYP51A, CYP51B*, and *CYP51C* (GenBank accession numbers MN296719, MN296724, and MN296725, respectively). Open boxes indicate ORF sequences, and grey boxes are upstream and downstream regions. Introns and their ORF positions are indicated with black lines. Bidirectional arrows underneath ORFs indicate the size of the entire ORF including introns.

**Figure 2:**
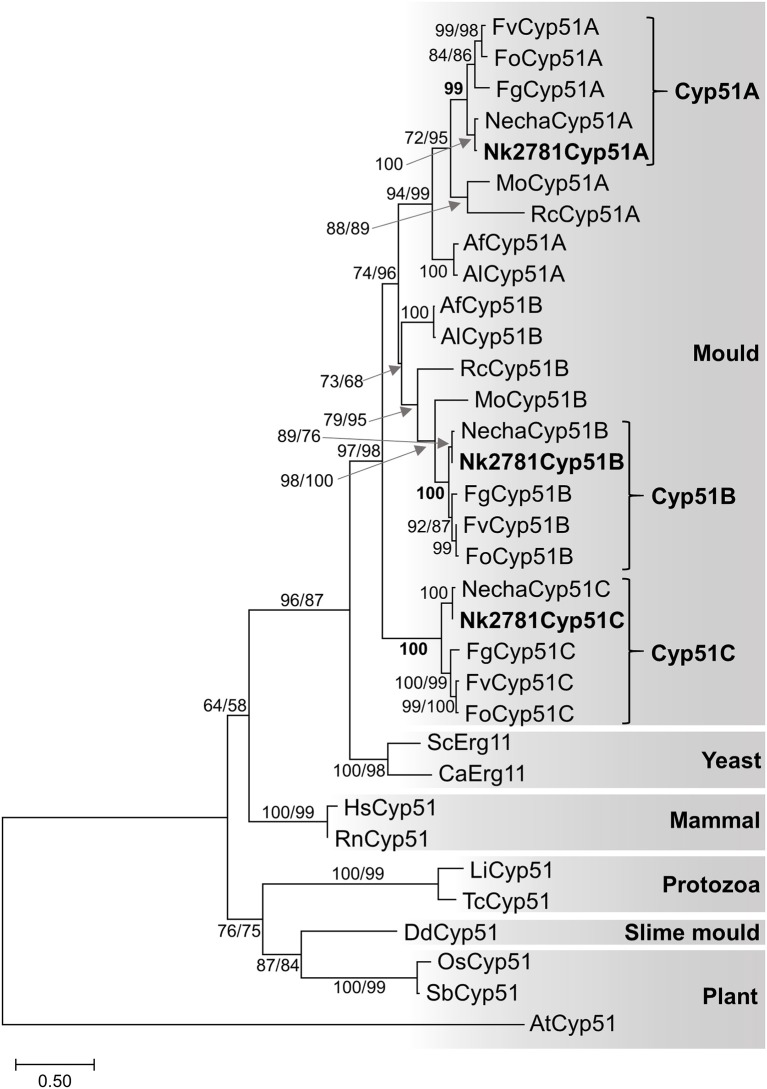
Maximum likelihood phylogram of Cyp51/Erg11 sequences of selected eukaryotes. Species names are abbreviated as: Af, *A. fumigatus*; Al, *A. lentulus*; Ca, *Candida albicans*; Fg, *F. graminearum*; Fo, *F. oxysporum*; Fv, *F. verticillioides*; Mo, *Magnaporthe oryzae*; Necha, *Nectria haematococca*; Rc, *Rhynchosporium commune*; Sc, *Saccharomyces cerevisiae*; protozoans Li, *Leishmania infantum* and Tc, *Trypanosoma cruzi*; slime mould Dd, *Dictyostelium discoideum*; mammals Hs, *Homo sapiens* and Rn, *Rattus norvegicus*; plants, AT *Arabidopsis thaliana*, Os, *Oryza sativa* and Sb, *Sorghum bicolor*. The *A. thaliana* Cyp51 sequence was used as the outgroup. Nk2781 Cyp51A, -B and -C are in bold letters. Numbers at internodes represent the percentage of maximum parsimony (MP) and maximum likelihood (ML) bootstrap support (MP-BS/ML-BS) of 1,000 replicates; a single number means both values were identical. The scale bar indicates the number of amino acid substitutions per position (the alignment contains 398 residues).

All further investigations were performed with *N. keratoplastica* because these isolates were the most frequently isolated FSSC species in the clinic (11 of 15 strains; 73%, Table 2). Using *N. haematococca* mpVI 77-13-4 *CYP51A, CYP51B*, and *CYP51C* sequences as a guide, we isolated and sequenced the three orthologous ORFs of *N. keratoplastica* Nk2781, including parts of their 5′ upstream and 3′ downstream sequences. *CYP51A, CYP51B*, and *CYP51C* of *N. keratoplastica* Nk2781 consisted of 1,583, 1,753, and 1,651 nucleotides, respectively (Figure 1). *CYP51A* had one intron (62 bp) and *CYP51B* (114 bp and 55 bp) and *CYP51C* (47 bp and 53 bp) each had two introns (Figure 1). All introns were verified by sequencing the ORFs cDNA. Although the intron positions were conserved relative to *N. haematococca*, their sizes varied slightly: intron 1 and intron 2 of *CYP51B* were 4 bp and 2 bp larger and intron 1 of *CYP51C* was 2 bp larger than their *N. haematococca* counterparts. As in *N. haematococca, CYP51A, CYP51B*, and *CYP51C* encoded proteins of 506, 527, and 516 amino acids, respectively. *N. keratoplastica* Cyp51A was 56% identical to Cyp51B and 47% identical to Cyp51C. The relationship of the three Nk2781 Cyp51 paralogues with those of other *Fusaria* species and with those from plants and mammals is illustrated in Figure 2. The three paralogues formed three distinct phylogenetic branches with their respective *N. haematococca, F. graminearum, F. oxysporum*, and *F. verticillioides* orthologs (Figure 2).

### A Conserved Leucine at the Entry Gate of Cyp51A in *Neocosmospora* and *Fusarium* spp Possibly Contributes to Long-Tailed Azole Resistance

**Figure 3:**
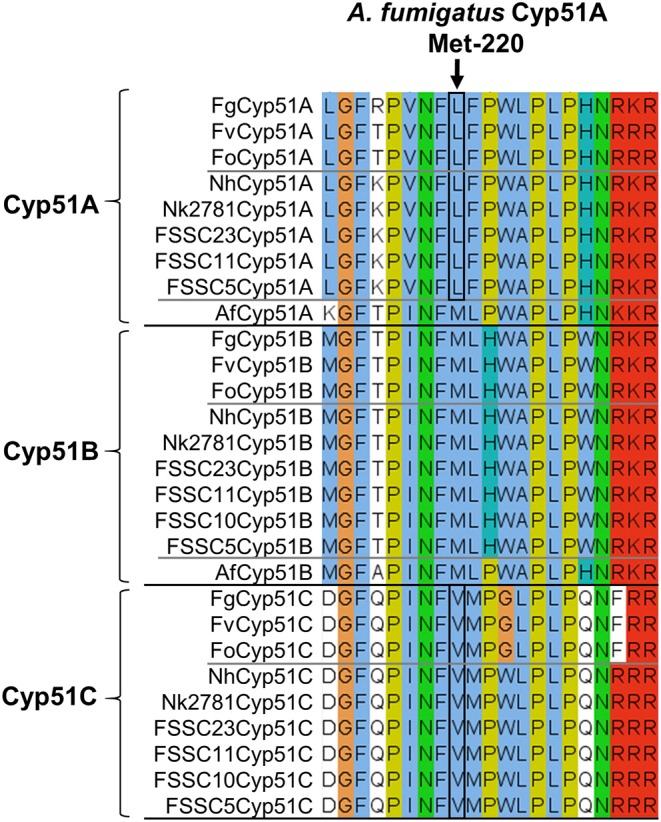
Alignment of Cyp51A, Cyp51B, and Cyp51C orthologs of three *Fusarium* species (top), six FSSC species including *N. keratoplastica* Nk2781 (centre) and *A. fumigatus* (it has only two Cyp51 orthologs, Cyp51A, and Cyp51B; bottom). FSSC10 also had only two Cyp51 orthologs, Cyp51B, and Cyp51C. The *A. fumigatus* Cyp51A-M220 equivalent residue is indicated with an arrow, and the *Fusarium* and FSSC equivalent Cyp51A-L and Cyp51C-V residues are encircled in black.

To identify possible changes in *CYP51A* that may explain the VRC resistance phenotype of group II *N. keratoplastica* isolates, we sequenced the entire ORF of eight clinical isolates: six group I strains, Nk994, Nk553, Nk1931, Nk620, Nk1049, and Nk2622 and two group II strains, Nk2309 and Nk2781. There were 12 non-synonymous SNPs, all in positions that have not been reported to be associated with azole resistance in ascomycetous fungi like *A. fumigatus* (Howard et al., 2009; Wiederhold et al., 2016; Moore et al., 2017) (see Supplementary Figure S3, Table S4). One notable exception—although obviously not the reason for VRC resistance because it was conserved across all eight *N. keratoplastica* isolates—was L218 in the equivalent position to M220 of *A. fumigatus* Cyp51A. Mutations of Cyp51A-M220 to I, K, T, or V residues were associated with azole resistance in *A. fumigatus* especially with resistance to the long-tailed azoles ITC and POS (Supplementary Table S4) (Howard et al., 2009; Wiederhold et al., 2016) and were predicted to affect the substrate entry gate (Snelders et al., 2010). The *N. keratoplastica* Cyp51A-L218 equivalent residues in *N. keratoplastica* Cyp51B and Cyp51C were M236 and V228, respectively. Interestingly, these three AfuCyp51A-M220 equivalent residues (L218, M236, and V228) were conserved across all three *Fusarium* and *Neocosmospora* Cyp51A, Cyp51B, and Cyp51C paralogs (Figure 3).

### A 23 bp *CYP51A* Promoter Deletion Was Tightly Associated With VRC Resistance in 9 Group II FSSC Isolates

**Figure 4:**
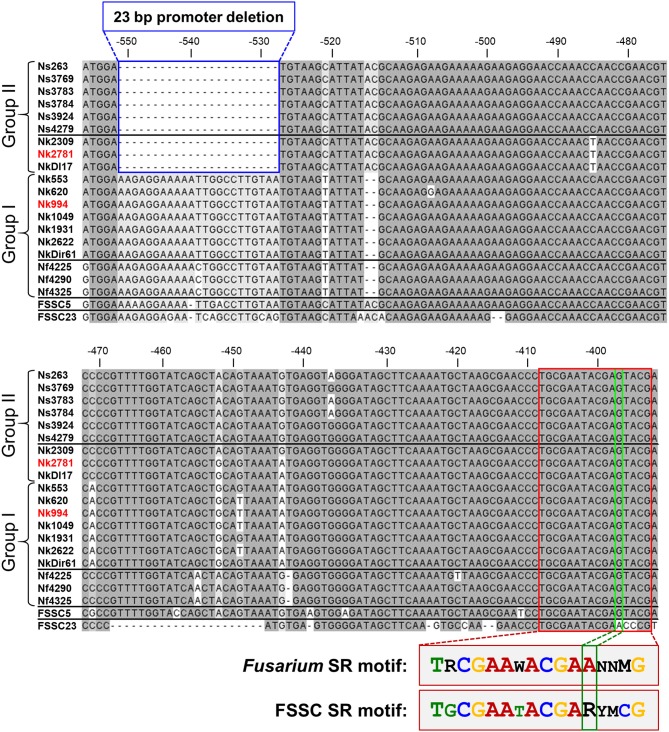
*CYP51A* promoter alignment (from −556 to −392 upstream of the Nk2781 *CYP51A* ATG start codon) of 19 FSSC isolates (6 *N. suttoniana*, 10 *N. keratoplastica*, 3 *N. falciformis*) and of FSSC5 and FSSC23 that were used as controls. The 23 bp *CYP51A* promoter deletion of FSSC isolates (Group II; 3 *N. keratoplastica*, 6 *N. suttoniana*) with high MIC_VRC_s (>32 mg/l) are bordered in blue. FSSC isolates (Group I; 7 *N. keratoplastica*, 3 *N. falciformis*) with lower MIC_VRC_s (≤12 mg/l) are underneath the group II sequences. The putative 16-bp sterol regulatory *cis*-element that was conserved in all FSSC isolates is bordered in red. There was one major change (bordered in green) to the core CGAA-NN-CGAA sterol regulatory (SR) binding motif of FgSR (Liu et al., 2019). FSSC23 had the conserved CGAA-NN-CGAA motif in the same position. A comparison of the conserved FSSC SR motif with the previously reported FgSR motif is provided underneath the alignment. The sequences of the red highlighted *N. keratoplastica* strains, Nk2781, and Nk994, were used for the VRC induction experiments.

Sequencing the *CYP51A* promoter from ten *N. keratoplastica* isolates revealed a 23 bp promoter deletion (from −551 to −528) that was only present in the three group II (MIC_VRC_ > 32 mg/l) isolates (Figure 4, Table 3). There were no other group II specific sequences within the first 750 nucleotides upstream of the *CYP51A* ATG start codon. To our surprise, we found the same 23 bp promoter deletion in all six *N. suttoniana* FSSC isolates as well, while the three *N. falciformis* FSSC isolates with MIC_VRC_s ≤ 4 mg/l had an almost identical 23 bp sequence as the seven group I *N. keratoplastica* isolates with MIC_VRC_s ≤ 12 mg/l (one nucleotide difference was observed at position −539 “T” in *N. keratoplastica* and a “C” in *N. falciformis*; Figure 4). Thus, the tight association of VRC resistance (MIC > 32 mg/l) with a 23 bp *CYP51A* promoter deletion was conserved across species boundaries and their origin of isolation; group I (MIC_VRC_s ≤ 12 mg/l) and group II (MIC_VRC_s > 32 mg/l) isolates contained both clinical as well as environmental FSSC isolates. The *CYP51A* promoter sequences of FSSC5 and FSSC23, retrieved from the Joint Genome Institute (JGI) database, aligned reasonably well with the sequences of the group I FSSC isolates. It is also important to note that there were other consistent mutations within the group II isolates: a “T” to “C” nucleotide change and two nucleotide “AC” insertion at positions −521 and −516 to −517, respectively. However, those mutations are less likely to be involved in the altered VRC susceptibility as they were also present in FSSC5 and FSSC23 *CYP51A* promoter sequences which did not carry the 23 bp deletion.

To explore the possibility that the 23 bp *CYP51A* promoter deletion of group II FSSC isolates affected a potentially important transcription factor (TF) binding site(s), we searched the entire *CYP51A* promoter for the recently identified TF binding site of the major sterol regulatory protein of *F. graminearum*, FgSR (Liu et al., 2019). FgSR is a zinc-cluster family TF that binds as a homodimer to a 16-bp *cis*-element containing two conserved CGAA repeats separated by 2 nucleotides (Figure 4). All 19 FSSC isolates sequenced had one conserved, although slightly modified (i.e., the distal CGAA repeat element was changed to CGAG in most FSSC species; Figure 4), 16 bp sterol regulatory binding site ~120 bp downstream (−409–393) of the 23 bp *CYP51A* promoter deletion (Figure 4).

### *CYP51A* and *CYP51B* Transcript Levels

**Figure 5:**
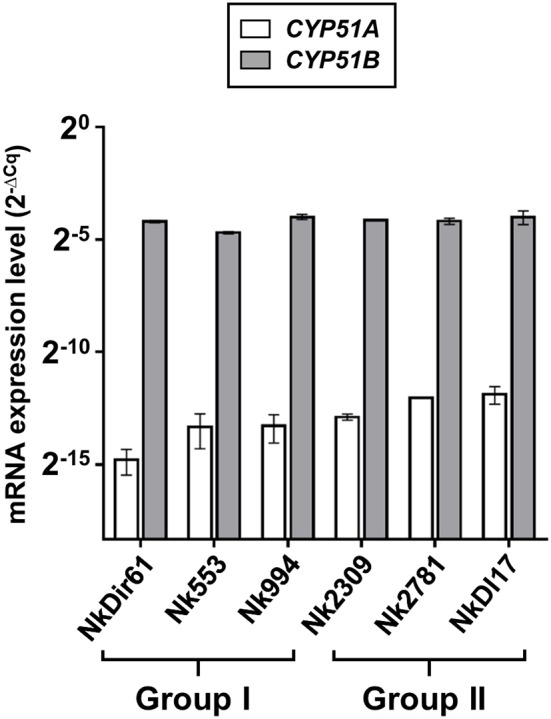
Quantification of *CYP51A* and *CYP51B* mRNA expression levels (2^−Δ*Cq*^) in logarithmic phase cells of six *N. keratoplastica* isolates (group I: NkDir61, Nk553, Nk994; group II: Nk2309, Nk2781, NkDI17). The fold differences between the *GPD1* normalised *CYP51B* and *CYP51A* mRNA levels and their group averages are listed in Table 4.

The amount of total RNA obtained from 50 ml *N. keratoplastica* cell cultures ranged from 120 to 400 μg (260/280 ratio: 1.83–2.01). The qPCR assay amplification efficiencies using cDNA as template and qPCR primers designed according to *N. keratoplastica* sequences were between 100 and 108% for all amplicons. We selected *GPD1* for transcript level normalisation (2^−Δ*Cq*^) and to calculate fold changes (2^−ΔΔ*Cq*^). Although generally much lower than *CYP51B*, the normalised *CYP51A* mRNA expression levels of the three group II strains were 1.3, 2.4, and 2.6 times higher than in the strain with the lowest MIC_VRC_ (1 mg/l), Nk994 (Figure 5). The highest *CYP51A* mRNA expression level was observed in strain NkDI17 (group II) which was 7.5 times higher than the lowest, in strain NkDir61 (group I). The normalised *CYP51B* transcript levels, however, hardly varied at all between the six strains (Figure 5). As expected, *N. keratoplastica CYP51B* mRNA levels were much higher than *CYP51A*. They were, on average, 859 and 295 (Table 4) times higher than *CYP51A* mRNA levels in the three group I and the three group II strains, respectively (Figure 5). However, the difference between the mean mRNA level fold differences (*CYP51B*/*CYP51A)* for each group was not statistically significant (*p*-value = 0.20).

**Table 4:** Fold differences between *GPD1* normalised *CYP51A* and *CYP51B* mRNA expression levels (2^−Δ*Cq*^) of logarithmic phase cells of six *N. keratoplastica* isolates.

	**Strain**	**Fold difference**		
		***CYP51B*** ***CYP51A***	**Mean**	***P*-value**
	NkDir61	1,550		
Group I	Nk553	400	859	
	Nk994	627		0.20
	Nk2309	431		
Group II	Nk2781	219	295	
	NkDI17	236		

*Individual CYP51A and CYP51B mRNA expression levels for each strain are presented in Figure 5*.

### VRC Induced *CYP51A* mRNA Expression ~6,500-Fold Reaching Levels Similar to *CYP51B*

**Figure 6:**
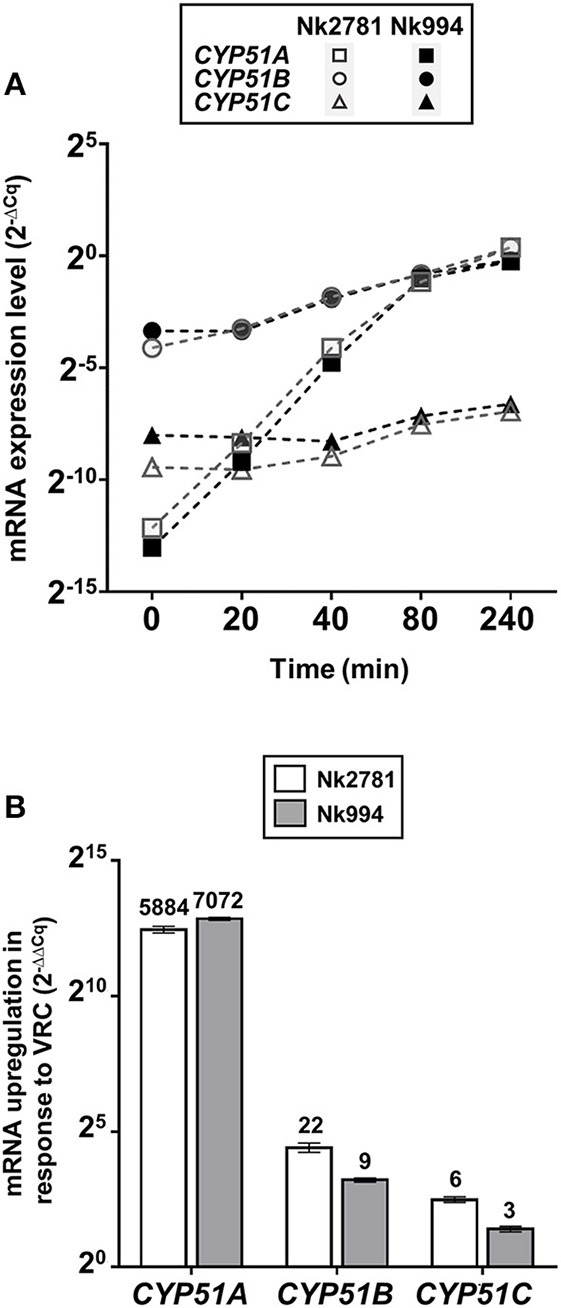
VRC induction of *CYP51A, CYP51B* and *CYP51C*. **(A)** *GPD1* normalised *CYP51A, CYP51B*, and *CYP51C* mRNA expression levels (2^−Δ*Cq*^) of Nk2781 (MIC_VRC_ > 32 mg/l) and Nk994 (MIC_VRC_ = 1 mg/l) *N. keratoplastica* strains harvested at the indicated times (0–240 min) after VRC induction (16 mg/l) of logarithmic phase cells grown for 21 h at 30°C in 50 ml PDB medium with shaking at 150 rpm. **(B)** Graph of the fold changes (2^−ΔΔ*Cq*^) of the *GPD1* normalised *CYP51A, CYP51B*, and *CYP51C* mRNA expression levels for both strains after 240 min of VRC induction relative to the expression levels of uninduced logarithmic cells at time zero. Numbers on top of the bars indicate fold changes.

The amount of total RNA obtained from 50 ml *N. keratoplastica* cell cultures (harvested at *t* = 0, 20, 40, 80 or 240 min) ranged from 9 to 241 μg (260/280 ratio: 1.95–2.01). At time zero of the VRC induction experiment, the *CYP51A* mRNA levels of logarithmic phase Nk2781 cells were 230 and 5 times lower than those of *CYP51B* and *CYP51C*, respectively, while *CYP51A* mRNA levels of Nk994 cells were 802 and 11 times lower, respectively (Figure 6A). However, after 80 min VRC exposure, *CYP51A* mRNA levels reached levels that were comparable to *CYP51B*, but they remained higher (1.6- to 2.2-fold) in Nk2781 than in Nk994 throughout the 240 min induction period, although they were slightly lower (1.3-fold) at the 80 min time point (Figure 6A). The transcript levels of *CYP51C* remained lower than *CYP51B* throughout the same 240 min time period (i.e., 40 times lower at time zero and 200 times lower at 240 min in Nk2781; 20 and 90 times lower in Nk994). Although all three *CYP51* paralogues of Nk2781 and Nk994 were induced by VRC, VRC induction was most pronounced for *CYP51A* (5,884 and 7,072-fold upregulation, respectively), less pronounced for *CYP51B* (22- and 9-fold upregulation, respectively), and even less so for *CYP51C* (6- and 3-fold upregulation, respectively; Figure 6B). Exposing logarithmic cells of the same two strains for 20 min to four different stress conditions, high salt (500 mM NaCl), high pH (pH 7.0), heat (37°C), and oxidative stress (2 mM H_2_O_2_), resulted in changes in *CYP51* mRNA expression. There was moderate downregulation of all three *CYP51* paralogues in both strains in response to salt (1.9- to 3.6-fold) and heat stress (1- to 15-fold). *CYP51A* transcript levels were increased (1.8- and 3-fold higher), however, when exposed to high pH. *CYP51C* transcript levels were particularly strongly affected (12- and 15-fold downregulation) by heat stress (Table 5). Exposing either *N. keratoplastica* strain to oxidative stress had little effect on all three *CYP51* paralogue expression levels (Table 5).

**Table 5:** Fold increased (+) or decreased (–) *GPD1* normalised *CYP51A, CYP51B*, and *CYP51C* mRNA levels of Nk2781 (MIC_VRC_ > 32 mg/l) and Nk994 (MIC_VRC_ = 1 mg/l) cells exposed for 20 min to the indicated stress conditions.

	**Fold-regulation**					
**Gene**	***CYP51A***		***CYP51B***		***CYP51C***	
**Stress/Strain**	**Nk2781**	**Nk994**	**Nk2781**	**Nk994**	**Nk2781**	**Nk994**
*Control*	1.00	1.00	1.00	1.00	1.00	1.00
*2 mM H_2_O_2_*	−1.18	−1.18	+1.35	+1.18	+1.08	−1.21
*500 mM NaCl*	−1.94	−2.95	−3.63	−3.04	−2.17	−2.09
*pH 7.0*	+1.83	+3.03	−1.58	−1.54	−2.44	−1.72
*37°C*	−3.02	−1.04	−2.85	−8.85	−15.46	−11.98

## Discussion

The 25 FSSC isolates from across Malaysia comprised 13 *N. keratoplastica*, six *N. suttoniana*, five *N. falciformis*, and one *N. petroliphila* isolate. *N. falciformis* strains were equally distributed among clinical (2) and environmental (3) isolates. However, most (11; 85%) *N. keratoplastica* and *N. petroliphila* strains were clinical isolates while most (6; 86%) *N. suttoniana* strains were of environmental origin. The species distribution of the 15 clinical FSSC isolates agreed with previous reports that identified *N. petroliphila* and *N. keratoplastica* (FSSC types 1 and 2) as the most frequent FSSC species isolated in the clinic (Chang et al., 2006; O'Donnell et al., 2012). *N. keratoplastica* was reported to cause invasive fusariosis in hematologic patients (Chiewchanvit et al., 2017), keratitis (Tupaki-Sreepurna et al., 2017), onychomycoses (Guevara-Suarez et al., 2016), and eumycetoma (Al-Hatmi et al., 2017a). It has also been reported to cause disease in equine and marine vertebrates and invertebrates (O'Donnell et al., 2016).

A high degree of agreement between the Etest, the EUCAST (96–100%), and the CLSI (90–100%) protocols for ITC, VRC, and AMB susceptibilities in *Fusarium*/*Neocosmospora* species has been reported (Tortorano et al., 2014; Al-Hatmi et al., 2017b). Although there are no clinical breakpoints for this fungal group, ECVs were established in 2016 (Espinel-Ingroff et al., 2016). The antifungal susceptibilities of our 25 FSSC isolates agreed with previous studies (Tortorano et al., 2014; Espinel-Ingroff et al., 2016) that reported high ITC (≥16 mg/l) and POS (≥8 mg/l) and variable VRC (0.5 to ≥16 mg/l) MICs for most FSSC isolates. The high (≥32 mg/l) MIC_VRC_s of 44% of the isolates [5 clinical (33%) and 6 environmental (60%)] is of serious concern given that VRC is a recommended drug for the treatment of IF (Pascual et al., 2008; Efe İris et al., 2016). AMB appears to be the only effective treatment option for IF (Espinel-Ingroff et al., 2016). Although two recently introduced imidazoles, luliconazole, and lanoconazole, have shown promising *in vitro* activities against various *Fusarium* and *Neocosmospora* isolates (Abastabar et al., 2018; Todokoro et al., 2019) their application appears limited to topical treatment of superficial fungal infections (Scher et al., 2014; Gupta and Daigle, 2016). Combination antifungal therapy is a potentially useful alternative for difficult-to-treat invasive mould infections. *In vitro* synergies between VRC and micafungin (Heyn et al., 2005), VRC and terbinafine (Córdoba et al., 2008), and VRC and AMB antifungal drug combinations (Ho et al., 2007) have been reported for various *Fusarium* species. In addition, the successful treatment of a patient with IF using a VRC and liposomal AMB combination was recently reported (Efe İris et al., 2016).

We found no *N. keratoplastica* Cyp51A mutations that were associated with high MIC_VRC_s (>32 mg/l) of group II isolates Nk2781 and Nk2309. However, the Cyp51A of all *N. keratoplastica* isolates (Supplementary Figure S3), including all other sequenced *Fusarium* and *Neocosmospora* FSSC species, had a conserved leucine in a position that was equivalent to M220 of *A. fumigatus* Cyp51A (Figure 3). Mutation of *A. fumigatus* Cyp51A-M220I caused ITC resistance and 4-fold increased MIC_VRC_s in two separate ITC resistant clinical *A. fumigatus* isolates (Chen et al., 2005; Snelders et al., 2010). Previous studies proposed that mutating M220 in a loop region near the substrate entry channel into the central binding cavity of Cyp51A may block the access of larger azoles like ITC with its long hydrophobic tail (Howard et al., 2009; Snelders et al., 2010). The *A. fumigatus* Cyp51A-G54 and -M220 equivalent residues lining the substrate/inhibitor entry channel in the crystal structure of *S. cerevisiae* Cyp51 (Monk et al., 2014) bound to ITC is depicted in Supplementary Figure S4. Perhaps the conserved L218 residue at the substrate entry channel of *N. keratoplastica* Cyp51A, and all other *Fusarium* and *Neocosmospora* FSSC species (Figure 3), is one of the reasons why these moulds do not respond well to azole antifungals with long hydrophobic tails (ITC and POS).

Most interesting, however, was the discovery of a 23 bp *CYP51A* promoter deletion in all nine group II (MIC_VRC_s > 32 mg/l) FSSC isolates (three *N. keratoplastica* and six *N. suttoniana*; Figure 4) that was absent in all ten group I (MIC_VRC_s ≤ 12 mg/l) FSSC isolates (seven *N. keratoplastica* and three *N. falciformis*). Remarkably, this tight association of the 23 bp *CYP51A* promoter deletion with the high VRC resistance phenotype of group II isolates reached across species boundaries and was independent of their clinical (e.g., Nk2781, NkDI17) or environmental (e.g., Nk994, NkDir61) origin (Figure 4). Similar promoter mutations resulted in pan-azole, including VRC, resistance (Wiederhold et al., 2016) in clinical *A. fumigatus* isolates that were possibly selected for by excessive agricultural use of azole antifungals (Chowdhary et al., 2013; Faria-Ramos et al., 2014; Vaezi et al., 2018). Unfortunately, we were not able to confirm an association between VRC resistance and the 23 bp *CYP51A* promoter deletion in all *N. falciformis* isolates, for technical reasons. Despite a number of efforts, we simply could not amplify the *CYP51A* promoter from the two clinical isolates (Nf0020 and Nf541) which showed high MIC_VRC_s > 32 mg/l. We suspect that their *CYP51A* 5′ upstream regions had sequence variations that prevented the primers designed against *N. haematococca CYP51A* to recognize their target sequence. Nevertheless, the tight association between VRC resistance and a 23 bp *CYP51A* promoter deletion preserved across species boundaries and independent of origin of isolation in the remaining 19 FSSC isolates was rather convincing. The collected evidence suggests two equally possible scenarios. Either the 23 bp *CYP51A* promoter deletion of group II FSSC isolates is a sequence variant of ancient origin (millions of years) that evolved before separation of individual FSSC species, or, alternatively, rare sexual recombination events between closely related FSSC species caused the exchange of the 23 bp *CYP51A* promoter deletion in the somewhat distant past (decades ago). Otherwise we would not expect to find the following species-specific SNPs: i) *N. falciformis* group I isolates had a C instead of a T inside the 23 bp promoter region that was deleted in group II isolates; ii) the three *N. keratoplastica* group II isolates had a unique T 45 bp downstream of the 23 bp promoter deletion, even though iii) they had identical sequences immediately surrounding the 23 bp promoter deletion with all other *N. suttoniana* group II isolates (Figure 4). Clearly, further investigations are necessary to distinguish between these two possibilities of: (i) an ancient *CYP51A* sequence variant shared between some closely related FSSC species, or (ii) a rather recent exchange of genetic material across species boundaries through rare sexual recombination events between closely related FSSC species that was perhaps selected for by the agricultural use of azoles over the past few decades.

Although VRC induced *CYP51A* mRNA expression in both Nk994 (group I) and Nk2781 (group II) to a similar extent, the *CYP51A* expression levels were consistently higher throughout the 4 h induction period (1.6- to 2.2-fold) in Nk2781 (Figure 6A). The ~6,500-fold increased *CYP51A* mRNA expression levels suggest that, like in *A. fumigatus* (Abastabar et al., 2019) and the plant fungal pathogen *F. graminearum* (Liu et al., 2011; Fan et al., 2013), *CYP51A* of *N. keratoplastica* is also a key player in the observed azole resistance phenotype of FSSC species. Sterol biosynthesis of Saccharomycotina species like *S. cerevisiae* or *C. albicans* is regulated by the sterol regulatory zinc-cluster TF Upc2 (Yang et al., 2015; Popp et al., 2017). Sterol biosynthesis of many other eukaryotes, including fungal species like *A. fumigatus, C. neoformans* and *Schizosaccharomyces pombe*, and also mammals including *Homo sapiens*, is regulated by a different type of sterol regulator: a TF called SREBP. A third type of sterol biosynthesis regulator, the zinc-cluster TF FgSR, has recently been discovered in the plant fungal pathogen *Fusarium graminearum* (Liu et al., 2019). This type of sterol regulatory network exists only in *Sordariomycetes* and *Leotiomycetes* fungi including *Neocosmospora* species of the FSSC. FgSR homodimers bind to a 16 bp *cis*-element of target gene promoters containing two conserved CGAA repeats. Sterol depletion triggers the activation of FgSR via MAP-kinase FgHog1 phosphorylation which, in turn, induces FgSR interaction with the chromatin remodelling complex SWI/SNF and the upregulation of target gene expression. As expected, we found an almost identical 16 bp *cis*-element ~400 bp upstream of the ATG start codon in the *CYP51A* promoter of all *Necosmospora* FSSC isolates and in all related *Fusarium* species. The only major difference was an A to G transition in the distal CGAA repeat (CGAG) that was conserved throughout the FSSC (Figure 4). However, the 16 bp *cis*-element was 120 bp downstream of the 23 bp deletion and present in all *Neocosmospora* isolates sequenced. We speculate that the 23 bp *CYP51A* promoter deletion affects *CYP51A*-chromatin-packing that causes a slightly (1.3–7.5-fold) increased basal *CYP51A* mRNA expression level in group II FSSC isolates (Figure 5). The presence of an unchanged sterol regulatory element in group II FSSC isolates explains why there was no significant difference in the regulation of *CYP51A* mRNA expression in group I and group II isolates in response to VRC.

Expression and/or upregulation of ATP-binding cassette (ABC) transporters is also frequently associated with azole resistance in fungi (Lamping et al., 2009; Panapruksachat et al., 2016; Watanasrisin et al., 2016; Paul et al., 2019). In *A. fumigatus*, the AtrR TF binds to, and regulates, both *cyp51A* and the ABC transporter *abcG1*. Binding of AtrR to a 34-bp tandem repeat element discovered in *cyp51A* promoters of azole resistant *A. fumigatus* clinical isolates increased *cyp51A* expression and azole resistance (Howard et al., 2009; Wiederhold et al., 2016; Paul et al., 2019). Whether the *CYP51A* promoter mutations alone caused ~2–32 times higher VRC MICs of group II strains or whether other factors (e.g., ABC transporters) also contribute to the observed azole resistance phenotype of FSSC isolates remains to be investigated. Nevertheless, our data suggest that the 23 bp *CYP51A* promoter deletion is a potentially valuable new VRC resistance marker to enable clinicians to choose the correct treatment option for the often lethal IF.

In summary, *N. keratoplastica CYP51A* mRNA levels are ~6,500-fold upregulated in response to azole antifungals to compensate for the loss of *CYP51B* function due to azole inhibition. There was a strong association of VRC resistance with a 23 bp *CYP51A* promoter deletion in all *Neocosmospora* FSSC isolates tested. The conserved “entry-gate” residue *N. keratoplastica* Cyp51A-L218 may possibly be related to the long-tailed azole resistance phenotype of FSSC species. A novel 16 bp sterol regulatory *cis*-element was present ~400 bp upstream of the ATG start codon of all sequenced *CYP51A* promoters. Taken together, our findings provide important first clues about possible azole resistance mechanisms in the medically, and agriculturally, important fungal pathogens of the FSSC.

## Data Availability Statement

DNA sequences generated in this study have been made publicly available at GenBank. The datasets for phylogenetic analysis will be made available by the authors upon request, without undue reservation, to any qualified researcher.

## Author Contributions

JJ performed the experiments and most of the data analysis. EL contributed to the experimental design and data analysis. JS, EL, and RC contributed to the conception and design of the study. LZ and MA provided the isolates used in this study and performed the morphological identification. TM supervised the qPCR experiments and mRNA expression analysis. JJ wrote the manuscript. EL, RC, TM, and JS contributed to manuscript revision. All authors read and approved the final manuscript.

### Conflict of Interest

The authors declare that the research was conducted in the absence of any commercial or financial relationships that could be construed as a potential conflict of interest.
