# Supplementary material for: A 23 bp cyp51A Promoter Deletion Associated With Voriconazole Resistance in Clinical and Environmental Isolates of Neocosmospora keratoplastica
Source: Front Microbiol. 2020 Mar 31;11:272. doi: 10.3389/fmicb.2020.00272 (PMC7136401; doi:10.3389/fmicb.2020.00272)
Supplement: Supplementary file 1 [file Data_Sheet_1.docx]

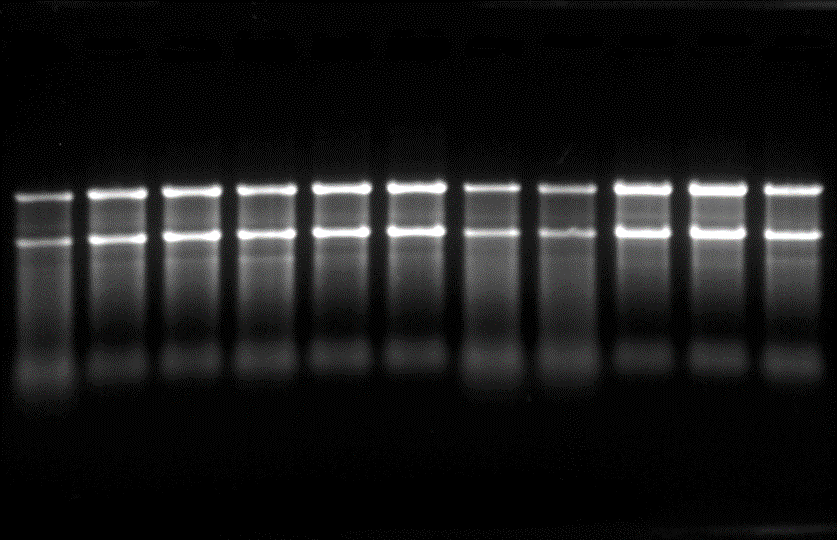


**1 2 3 4 5 6 7 8 9 10**


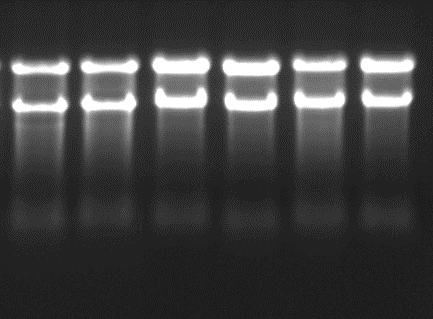


**1 2 3 4 5 6**

**B**

**A**

**Supplementary Figure S1.** RNA gel electrophoresis of total RNA (1 µg). Total RNA was isolated from logarithmic phase cells of **(A)** NkDir61, Nk553, Nk994, Nk2309, Nk2781 and NkDI17 (lanes 1 to 6, respectively) grown in 50 ml PDB for 21 h at 30°C and **(B)** Nk2781 (lane 1 to 5) and Nk994 (lane 6 to 10) grown in 50 ml PDB in the presence of 16 mg/l VRC for an additional 0, 20, 40, 80, and 240 min. RNA was visualised with EtBr staining. **RNA gel components:** 1.2% agarose gel in 1X MOPS and 1.11% formaldehyde; 50 V; 80 min; 1X MOPS running buffer: 20 mM MOPS, 5 mM NaOAc, 1 mM EDTA; 1 µg total RNA in a minimum of 5 µl combined with equal volume of freshly made sample buffer (1X MOPS buffer, 7.4% formaldehyde, 50% formamide and 0.01 mg/ml EtBr).


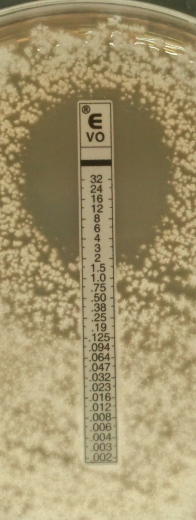

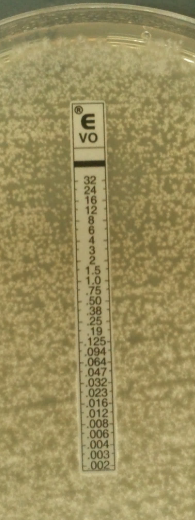

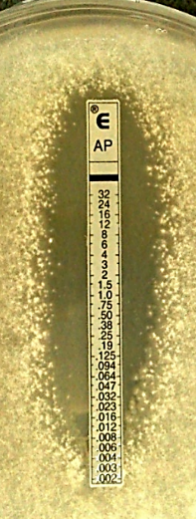

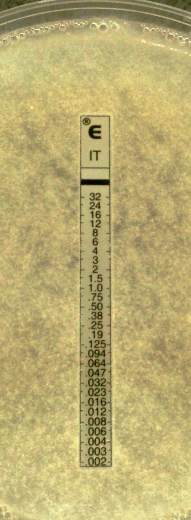

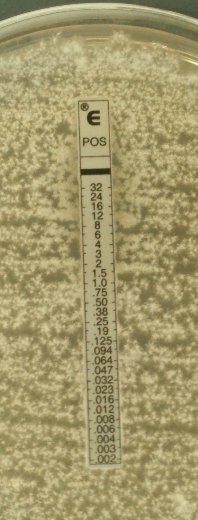

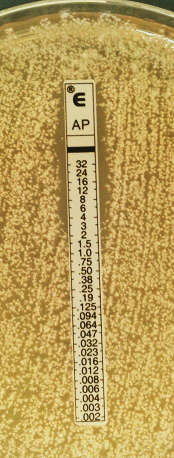


**Nk2781**

**Np667**

**Ns3769**

**Nk2781**

**Ns3873**

**Nf541**

**A**

**B**

**C**

**D**

**E**

**F**

**ITC**

**POS**

**VRC**

**AMB**

**Supplementary Figure S2.** Representative Etest results for various *Neocosmospora* isolates. All strains showed ITC and POS MICs > 32 mg/l [e.g. Nk2781 **(A)** and Ns3769 **(B)**]; Np667 and Nk2781 MIC_VRC_s were 2 mg/l **(C)** and > 32 mg/l **(D)**, respectively; Nf541 and Ns3873 MIC_AMB_s were 0.023 mg/l **(E)** and 3 mg/l **(F)**, respectively.


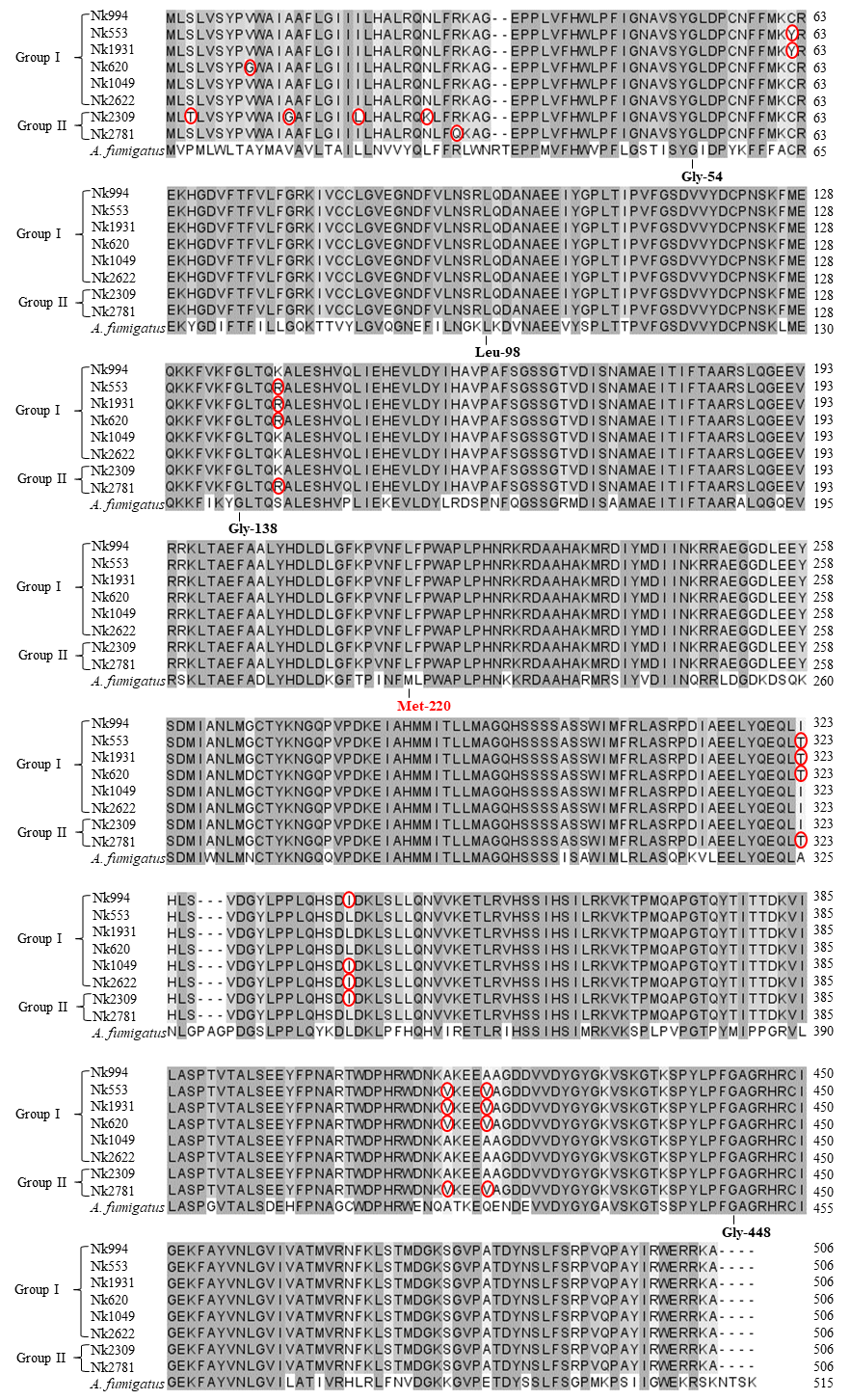


**Tyr-121**

**Thr-289**

**Supplementary Figure S3.** Alignments of eight *N. keratoplastica* Cyp51A (506 amino acids) sequences with *A. fumigatus* Cyp51A, AF338659 (515 amino acids). Twelve SNPs that differed between the eight sequences are encircled in red. Residues for which mutations have been associated with azole resistance in *A. fumigatus* (Supplementary Table S4) are indicated. Group I: MIC_VRC_s = 1.5 - 12 mg/l; Group II: MIC_VRC_s > 32 mg/l.

**Supplementary Figure S4.** Graphical illustration of the *A. fumigatus* Cyp51A-G54 and -M220 equivalent residues lining the substrate/inhibitor entry channel in the crystal structure of *S. cerevisiae* Cyp51 bound to itraconazole (Monk et al., 2014). The two entry-gate residues of *S. cerevisiae* (Sc) G73 and V242, equivalent to *A. fumigatus* (Af) G54 and M220, are shown as red sticks and their distances to the hydrophobic itraconazole tail (grey dotted lines) are shown above in grey. The coordination of the nitrogen (blue) of the imidazole ring of itraconazole with the centrally located heme iron is indicated with a black dotted line. The ribbon model of ScCyp51 is shown in rainbow colours starting from the N-terminus in blue and finishing at the C-terminus in red. The centrally located heme is shown as black sticks and itraconazole is represented with coloured sticks (C = magenta; N = blue; O = red; H = grey). The lipid bilayer of the endoplasmic reticulum that is traversed by the N-terminal transmembrane anchor of ScCyp51 is indicated light blue.

**Supplementary Table S1.** DNA oligomer primers used for PCR, qPCR and DNA sequencing.

| **Name^a, b^** | **Sequence (5’ to 3’)** |
| --- | --- |
| *CYP51A* | |
| Nh*CYP51A*-(–944)-F | ATCGCAAACAGCCAACTGGA |
| Nk*CYP51A*-133-R | TACGACACAGCATTGCCGAT |
| Nh*CYP51A*-(–63)-F | TAAAAGCCCTCCGTGTTCCTC |
| Nh*CYP51A*-849-R | GTGCAACCCATCAAGTTGGC |
| Nk*CYP51A*-701-F | GACCTTGATCTTGGCTTCAAG |
| Nh*CYP51A*-(+71)-R | TCTAGAATGCCAATAGTAGA |
| Nk*CYP51A*-983-F* | CCTGGATCATGTTCCGTCTT |
| Nk*CYP51A*-1076-R* | GAAGGGAGAGCTTGTCGATG |
| *CYP51B* | |
| Nh*CYP51B*-(–29)-F | GCTCTCAACAGCAACAACCTTCTAC |
| Nh*CYP51B*-623-F | AGCCCATGATAGTTCATGAAGATCG |
| Nh*CYP51B*-(+239)-R | TCCAAGACACACATCATCTCATTCC |
| Nk*CYP51B*-785-F* | TCTTCACCGCCTCCCACGCCC |
| Nk*CYP51B*-993-R* | GATGCTTCATCATGTCGTGCT |
| *CYP51C* | |
| Nh*CYP51C*-(–69)-F | AAACGAATGATGCTGACAGTCTGAC |
| Nh*CYP51C*-635-F | TCACCATCTACACCGCTTCCGGCTC |
| Nh*CYP51C*-(+113)-R | CGGCGAGAGGTTCAATTCCGATTAG |
| Nk*CYP51C*-872-F* | CGACATGATCTGGACCCTTATG |
| Nk*CYP51C*-934-R* | GCTGACCACCCATGAGAATAG |
| *ACT1* | |
| Nh*ACT1*-554-F | GTAAGTTTGATATTTGCGACACCTC |
| Nh*ACT1*-(+142)-R | TAAATATGAGATTCGACCATGATGC |
| Nk*ACT1*-1309-F* | ACGTCACCACCTTCAACTCC |
| Nk*ACT1*-1429-R* | GTCGGAGAGACCAGGGTACA |
| *GPD1* | |
| Nh*GPD1*-(–264)-F | CATGAGAGCTCTAGCTCCTGTTTAG |
| Nh*GPD1*-1126-R | CTTGAGCGATCTACGCTTACTTGGA |
| Nk*GPD1*-829-F* | CGAGAAGGGTGCTTCTTACGACGAG |
| Nk*GPD1*-1050-R* | AGTAACCCCACTCGTTGTCGTACCA |

**^a^** Nh = *N. haematococca*; Nk = *N. keratoplastica*.

**^b^** Numbers indicate the ORF position to which the 3’ end of the indicated primer binds; primers binding at the indicated positions upstream or downstream of an ORF are marked with a minus (upstream) or plus sign (downstream) placed in brackets.

* Primers used for qPCR.

**Supplementary Table S2. List of** DNA sequences and their GenBank accession numbers.

|  | **GenBank accession number^a^** | | | | |
| --- | --- | --- | --- | --- | --- |
| **Strain** | ***CYP51A*** | ***CYP51B*** | ***CYP51C*** | ***ACT1*** | ***GPD1*** |
| Nf541 | MN640610** | MN640620 | ꟷ | ꟷ | ꟷ |
| Nf4225 | MN640611* | MN640621 | ꟷ | ꟷ | ꟷ |
| Nf4290 | MN640612* | ꟷ | ꟷ | ꟷ | ꟷ |
| Nf4325 | MN640613* | ꟷ | ꟷ | ꟷ | ꟷ |
| Nk553 | MN296712 | MN296722 | ꟷ | MN296726 | MN296728 |
| Nk620 | MN296713 | ꟷ | ꟷ | ꟷ | ꟷ |
| Nk994 | MN296714 | ꟷ | ꟷ | ꟷ | ꟷ |
| Nk1049 | MN296715 | ꟷ | ꟷ | ꟷ | ꟷ |
| Nk1931 | MN296716 | ꟷ | ꟷ | ꟷ | ꟷ |
| Nk2309 | MN296717 | MN296723 | ꟷ | MN296727 | MN296729 |
| Nk2622 | MN296718 | ꟷ | ꟷ | ꟷ | ꟷ |
| Nk2781 | MN296719 | MN296724 | MN296725 | ꟷ | ꟷ |
| NkDI17 | MN296720* | ꟷ | ꟷ | ꟷ | ꟷ |
| NkDir61 | MN296721* | ꟷ | ꟷ | ꟷ | ꟷ |
| Ns263 | MN640614 | ꟷ | ꟷ | ꟷ | ꟷ |
| Ns3769 | MN640615 | ꟷ | ꟷ | ꟷ | ꟷ |
| Ns3783 | MN640616* | ꟷ | ꟷ | ꟷ | ꟷ |
| Ns3784 | MN640617 | ꟷ | ꟷ | ꟷ | ꟷ |
| Ns3924 | MN640618 | ꟷ | ꟷ | ꟷ | ꟷ |
| Ns4279 | MN640619* | ꟷ | ꟷ | ꟷ | ꟷ |

**^a^** ꟷ indicates not sequenced.

* Only the promoter region was sequenced.

** Sequencing of promoter region was unsuccessful.

**Supplementary Table S3.** List of the Cyp51 sequences that were used for the phylogenetic analyses in Figure 2 and their GenBank accession numbers.

|  | **GenBank accession number^a^** | | |
| --- | --- | --- | --- |
| **Organism** | **Cyp51A^b^** | **Cyp51B** | **Cyp51C** |
| Plant |  |  |  |
| *Arabidopsis thaliana* | AAB86510 | ꟷ | ꟷ |
| *Sorghum bicolor* | AAC49659 | ꟷ | ꟷ |
| *Oryza sativa* | XP_015617432 | ꟷ | ꟷ |
| Slime mould |  |  |  |
| *Dictyostelium discoideum* | XP_001134568 | ꟷ | ꟷ |
| Protozoa |  |  |  |
| *Trypanosoma cruzi* | AAP33132 | ꟷ | ꟷ |
| *Leishmania infantum* | ABM89546 | ꟷ | ꟷ |
| Mammal |  |  |  |
| *Homo sapiens* | AAB39951 | ꟷ | ꟷ |
| *Rattus norvegicus* | NP_037073 | ꟷ | ꟷ |
| Yeast |  |  |  |
| *Saccharomyces cerevisiae* | NP_011871 | ꟷ | ꟷ |
| *Candida albicans* | BAB03399 | ꟷ | ꟷ |
| Mould |  |  |  |
| *Aspergillus fumigatus* | AAK73659 | AAK73660 |  |
| *Aspergillus lentulus* | ADI80344 | AEU08387 |  |
| *Magnaporthe oryzae* | XP_362183 | XP_361987 |  |
| *Rhynchosporium commune* | AIF79454 | AIF79460 |  |
| *F. graminearum* | XP_011321548 | XP_011316750 | XP_011325340 |
| *F. oxysporum* | XP_018249824 | XP_018232321 | XP_018252292 |
| *F. verticillioides* | XP_018757408 | XP_018743733 | XP_018760287 |
| *Nectria haematococca* | XP_003045204 | XP_003054236 | XP_003051421 |

**^a^** ꟷ indicates a Cyp51 paralog that is absent in that species.

**^b^** Species with only one Cyp51 protein are listed in the Cyp51A column.

**Supplementary Table S4.** Amino acid changes and promoter mutations in *A. fumigatus* and *N. keratoplastica* *CYP51A* associated with azole resistance.

| ***A. fumigatus*** | | | |
| --- | --- | --- | --- |
| **Amino acid position^a^** | **Mutation** | **Azole resistance^b^** | **References** |
| Gly-54 | Glu, Lys, Arg, Val, Tyr | ITC, POS | (Howard et al. 2009; Wiederhold et al., 2016) |
| TR34/Leu-98 | Leu-98🡪His | Pan-azole | (Howard et al. 2009; Wiederhold et al., 2016) |
| TR46/Tyr-121/Thr-289 | Tyr-121🡪Phe,  Thr-289🡪Ala | Pan-azole & Isavuconazole | (Wiederhold et al., 2016; Moore et al., 2017) |
| Met-220 | Ile, Lys, Thr, Val | ITC, POS, variable VRC | (Howard et al. 2009; Wiederhold et al., 2016) |
| Gly-138 | Cys, Ser | Pan-azole | (Howard et al. 2009; Wiederhold et al., 2016) |
| Gly-448 | Ser | Pan-azole | (Howard et al. 2009; Wiederhold et al., 2016) |
| ***N. keratoplastica*** | | | |
| **Nucleotide position** | **Mutation** | **Azole resistance** | **References** |
| -551-528 | 23 Ns - ∆23 | VRC | This study |
|  |  |  |  |

**^a^** TR34 and TR46 are 34 and 46 bp tandem repeat promoter insertions in *A. fumigatus* *cyp51A*.

**^b^** ITC, itraconazole; POS, posaconazole; VRC, voriconazole.
